# Supplementary material for: Sniffin’ sticks smell identification test: exploring measurement properties in an adult Brazilian healthcare professionals sample
Source: PeerJ. 2025 Oct 28;13:e19733. doi: 10.7717/peerj.19733 (PMC12577572; doi:10.7717/peerj.19733)
Supplement: Supplemental Information 2 [file peerj-13-19733-s002.doc]

STROBE Statement—Checklist of items that should be included in reports of ***cross-sectional studies***

|  | Item No | Recommendation |
| --- | --- | --- |
| **Title and abstract** | 1 | (*a*) Indicate the study’s design with a commonly used term in the title or the abstract – **page 1, line 1** |
|  |  | (*b*) Provide in the abstract an informative and balanced summary of what was done and what was found – **page 1, line 27 - 52** |
| Introduction | | |
| Background/rationale | 2 | Explain the scientific background and rationale for the investigation being reported– **page 2, line 54 - 72** |
| Objectives | 3 | State specific objectives, including any prespecified hypotheses – **page 2, line 72 - 75** |
| Methods | | |
| Study design | 4 | Present key elements of study design early in the paper – **page 2, line 78-83** |
| Setting | 5 | Describe the setting, locations, and relevant dates, including periods of recruitment, exposure, follow-up, and data collection – **page 2, line 85 - 90** |
| Participants | 6 | (*a*) Give the eligibility criteria, and the sources and methods of selection of participants – **page 2, line 90 - 94** |
| Variables | 7 | Clearly define all outcomes, exposures, predictors, potential confounders, and effect modifiers. Give diagnostic criteria, if applicable – **not applicable** |
| Data sources/ measurement | 8* | For each variable of interest, give sources of data and details of methods of assessment (measurement). Describe comparability of assessment methods if there is more than one group – **page 2 and 3, lines 98 - 126** |
| Bias | 9 | Describe any efforts to address potential sources of bias – **page 3, lines 107 - 108** |
| Study size | 10 | Explain how the study size was arrived at – **page 2, line 85 - 90** |
| Quantitative variables | 11 | Explain how quantitative variables were handled in the analyses. If applicable, describe which groupings were chosen and why – **page 3, line 129-234** |
| Statistical methods | 12 | (*a*) Describe all statistical methods, including those used to control for confounding – **page 3 and 4, line 136 - 186** |
|  |  | (*b*) Describe any methods used to examine subgroups and interactions – **page 4, line 164 - 174** |
|  |  | (*c*) Explain how missing data were addressed – Addressing missing data is a crucial aspect of data analysis, as it can significantly affect the results and interpretations of a study. However, in this study, the issue of missing data was largely mitigated because the population was drawn from a hospital setting. This controlled environment allowed for more comprehensive data collection, ensuring that the data were complete and reducing the likelihood of missing information. As a result, we were able to maintain a high level of data integrity throughout the analysis. Thus, missing data was not addressed |
|  |  | (*d*) If applicable, describe analytical methods taking account of sampling strategy – **not applicable** |
|  |  | (*e*) Describe any sensitivity analyses – **not applicable** |
| Results | | |
| Participants | 13* | (a) Report numbers of individuals at each stage of study—eg numbers potentially eligible, examined for eligibility, confirmed eligible, included in the study, completing follow-up, and analysed– **page 4, line 190 - 194** |
|  |  | (b) Give reasons for non-participation at each stage– **page 4, line 190 - 194** |
|  |  | (c) Consider use of a flow diagram– **not applicable** |
| Descriptive data | 14* | (a) Give characteristics of study participants (eg demographic, clinical, social) and information on exposures and potential confounders– **page 5, line 235 -253** |
|  |  | (b) Indicate number of participants with missing data for each variable of interest– **not applicable** |
| Outcome data | 15* | Report numbers of outcome events or summary measures – **page 5, line 255 -260** |
| Main results | 16 | (*a*) Give unadjusted estimates and, if applicable, confounder-adjusted estimates and their precision (eg, 95% confidence interval). Make clear which confounders were adjusted for and why they were included – **page 5, line 235 -253** |
|  |  | (*b*) Report category boundaries when continuous variables were categorized – **page 4, line 194 - 202** |
|  |  | (*c*) If relevant, consider translating estimates of relative risk into absolute risk for a meaningful time period – **not applicable** |
| Other analyses | 17 | Report other analyses done—eg analyses of subgroups and interactions, and sensitivity analyses – **not applicable** |
| Discussion | | |
| Key results | 18 | Summarise key results with reference to study objectives – **page 6 , line 262 - 281** |
| Limitations | 19 | Discuss limitations of the study, considering sources of potential bias or imprecision. Discuss both direction and magnitude of any potential bias – **page 6, line 294 - 308** |
| Interpretation | 20 | Give a cautious overall interpretation of results considering objectives, limitations, multiplicity of analyses, results from similar studies, and other relevant evidence – **page 6 and 7, line 300 - 327** |
| Generalisability | 21 | Discuss the generalisability (external validity) of the study results **page 6, lines 271 - 286** |
| Other information | | |
| Funding | 22 | Give the source of funding and the role of the funders for the present study and, if applicable, for the original study on which the present article is based – **page 7, line 345 - 350** |

*Give information separately for exposed and unexposed groups.

**Note:** An Explanation and Elaboration article discusses each checklist item and gives methodological background and published examples of transparent reporting. The STROBE checklist is best used in conjunction with this article (freely available on the Web sites of PLoS Medicine at http://www.plosmedicine.org/, Annals of Internal Medicine at http://www.annals.org/, and Epidemiology at http://www.epidem.com/). Information on the STROBE Initiative is available at www.strobe-statement.org.
